# A yeast expression system for functional and pharmacological studies of the malaria parasite Ca^2+^/H^+^ antiporter

**DOI:** 10.1186/1475-2875-11-254

**Published:** 2012-08-01

**Authors:** J Enrique Salcedo-Sora, Steve A Ward, Giancarlo A Biagini

**Affiliations:** 1 Liverpool School of Tropical Medicine, Pembroke place, Liverpool L3 5QA, UK

**Keywords:** Calcium, Magnesium, Manganese, Malaria, Yeast, *Plasmodium*, Ca^2+^/H^+^ antiporter, *Saccharomyces cerevisiae*, Vacuole

## Abstract

**Background:**

Calcium (Ca^2+^) signalling is fundamental for host cell invasion, motility, *in vivo* synchronicity and sexual differentiation of the malaria parasite. Consequently, cytoplasmic free Ca^2+^ is tightly regulated through the co-ordinated action of primary and secondary Ca^2+^ transporters. Identifying selective inhibitors of Ca^2+^ transporters is key towards understanding their physiological role as well as having therapeutic potential, therefore screening systems to facilitate the search for potential inhibitors are a priority. Here, the methodology for the expression of a Calcium membrane transporter that can be scaled to high throughputs in yeast is presented.

**Methods:**

The *Plasmodium falciparum* Ca^2+^/H^+^ antiporter (PfCHA) was expressed in the yeast *Saccharomyces cerevisiae* and its activity monitored by the bioluminescence from apoaequorin triggered by divalent cations, such as calcium, magnesium and manganese.

**Results:**

Bioluminescence assays demonstrated that PfCHA effectively suppressed induced cytoplasmic peaks of Ca^2+^, Mg^2+^ and Mn^2+^ in yeast mutants lacking the homologue yeast antiporter Vcx1p. In the scalable format of 96-well culture plates pharmacological assays with a cation antiporter inhibitor allowed the measurement of inhibition of the Ca^2+^ transport activity of PfCHA conveniently translated to the familiar concept of fractional inhibitory concentrations. Furthermore, the cytolocalization of this antiporter in the yeast cells showed that whilst PfCHA seems to locate to the mitochondrion of *P. falciparum*, in yeast PfCHA is sorted to the vacuole. This facilitates the real-time Ca^2+^-loading assays for further functional and pharmacological studies.

**Discussion:**

The functional expression of PfCHA in *S. cerevisiae* and luminescence-based detection of cytoplasmic cations as presented here offer a tractable system that facilitates functional and pharmacological studies in a high-throughput format. PfCHA is shown to behave as a divalent cation/H^+^ antiporter susceptible to the effects of cation/H^+^ inhibitors such as KB-R7943. This type of gene expression systems should advance the efforts for the screening of potential inhibitors of this type of divalent cation transporters as part of the malaria drug discovery initiatives and for functional studies in general.

**Conclusion:**

The expression and activity of the PfCHA detected in yeast by a bioluminescence assay that follows the levels of cytoplasmic Ca^2+^ as well as Mg^2+^ and Mn^2+^ lend itself to high-throughput and quantitative settings for pharmacological screening and functional studies.

## Background

Ca^2+^ signalling drives a myriad of events in the *Plasmodium. falciparum* life cycle. They include erythrocyte invasion [1-3], *in vivo* synchronicity in the erythrocytic cycle [4], together with sexual differentiation, motility and invasion by ookinetes and sporozoites in the mosquito vector [5-7]. As in any eukaryote the parasite’s concentration of cytosolic free Ca^2+^ is tightly maintained at 50-150 nM [8,9]. In eukaryotes this is achieved by its active sequestration into various organelles and/or extrusion to extracellular space. Transporters that could mediate this activity in *P. falciparum* include two Ca^2+^ ATPases, a low-affinity transporter PfATP4 [10] and a higher affinity SERCA-like Ca^2+^ ATPase PfATP6 [11,12]. Intracellular Ca^2+^ in *Plasmodium* has been found in acidic compartments (e.g. food vacuole with a calculated free Ca^2+^ of 0.4-2 μM) [9,13,14]. Ca^2+^ sequestration has also been observed in the malaria parasite’s mitochondrion [15,16]. Besides Ca^2+^ pumps, low-affinity secondary transporters that facilitate the membrane transport of Ca^2+^ and other divalent cations (e.g. Mg^2+^, Mn^2+^) into organelles or through plasma membrane using a proton (in lower eukaryotes and plants) gradient in the opposite direction (Ca^2+^/H^+^ exchangers or antiporters) are known to mediate the dissipation of cytoplasmic peaks of Ca^2+^[17,18]. In this context a *P. falciparum* Ca^2+^/H^+^ antiporter (PfCHA) homologue to the family of CAtion eXchangers (CAX, Transporter Classification Database 2.A.19.2) [19] has been reported and characterized in oocytes of *X. laevis* as a divalent cation (Ca^2+^, Mn^2+^ and possibly Mg^2+^)/H^+^ exchanger [20].

*Saccharomyces cerevisiae* is a highly developed and widely used model organism. Furthermore, *S. cerevisiae* has become a model for eukaryotic Ca^2+^ homeostasis [21,22]. In the present work, PfCHA has been expressed in the yeast *vcx1Δ* (VaCuolar Ca^2+^/H^+^ eXchanger) gene knock-out mutant. A bioluminescence apoaequorin reporter system has been used to allow the detection of cytoplasmic Ca^2+^ in *vcx1Δ* where PfCHA is shown to be able to re-establish Ca^2+^ mobilisation from cytoplasm. In the apoaequorin system aequorin catalyses the oxidation of an imidazolopyrazinone (coelenterazine) upon Ca^2+^ binding and light is emitted from the oxidized and excited state of this chromophore that exists tightly bound to aequorin. *In vitro*, Ca^2+^ binding to apoaequorin triggers the emission of blue light (469 nm), although other activating cations such as trivalent lanthanides [23] and divalent Mn^2+^ and Mg^2+^ are also known to bind aequorin via three EF-hand Ca^2+^-binding sites [24].

Interestingly, whilst in the parasite PfCHA is reported to be sorted to the mitochondrion [20], in *S. cerevisiae* the exchanger is sorted to the vacuole. This finding offers further practical advantages for the studies of a membrane transporter such as PfCHA since yeast vacuoles are their main Ca^2+^ storage compartments. Yeast is an attractive organism for recombinant protein production as it combines highly developed genetic systems and ease of use with reductions in time and costs. Moreover, due to the challenging nature of expressing functional membrane proteins a yeast expression system for PfCHA is a valuable tool for further functional studies and pharmacological screens. To this extent, the 96-well format was used to further demonstrate divalent cation (i.e. Ca^2+^, Mg^2+^, Mn^2+^) transport by PfCHA in *vcx1Δ* yeast cells and present an inhibition assay with a cation antiporter inhibitor as a proof of concept of the opportunities offered by this expression system for the search of PfCHA inhibitors.

## Methods

### Gene cloning

Total RNA from *P. falciparum* 3D7 was extracted with Trizol (Invitrogen) following manufacturer’s protocol using parasites harvested from standard cultures [25]. Gene sequences used as reference were downloaded from PlasmoDB5.3 [26] and GenBank [27]. The polymerase chain reaction (PCR) product from total RNA for PFF0170w (PfCHA) was cloned in the pCRII-Topo vector (Invitrogen), and subcloned in the shuttle vectors of the pGREG series [28] between *Not*I and *Bam*HI restriction sites obtained from Euroscarf. Primers for PFF0170w were 5’-ATGGTTATGGGTAGAGTTC and 5’-TTATGATGTATCAAACCAG. The *S. cerevisiae VCX1* gene was directly cloned into pGREG505 using the following primers: 5’-gaattcgatatcaagcttatcgataccgtcgacaGGCTGCTGATAGCAAATAAA and 5’- gcgtgacataactaattacatgactcgaggtcgacGAATTTCTGCGCTACTGTTC. pGREG505 vector has the *LEU2* selectable marker and the *GAL1* promoter. *Escherichia coli* TOP10 cells (Invitrogen) were routinely used as recombinant plasmids host. For bioluminescence measurements of cytosolic Ca^2+^ yeast cells were transformed with a plasmid carrying the aequorin gene [29]. This gene cloned in a pSEY8 plasmid under *ADH3* promoter was kindly provided by David M. Bedwell (University of Alabama at Birmingham). Reverse transcription PCR and real-time PCR with iQ SYBR Green Supermix (Bio-Rad) in yeast were performed using total RNA extracted with RiboPure-Yeast (Ambion/Applied Biosystems, Austin, TX, USA) following manufacturer's instructions. cDNA was synthesized using ThermoScript (Invitrogen), and PCR amplifications using Platinum Taq High Fidelity (Invitrogen).

### Yeast strains and media

Yeast *Saccharomyces cerevisiae* haploid strain Y03825 (MATa,*his3Δ1,leu2Δ0,met15Δ0,ura3Δ0,YDL128w::kanMX4*) was obtained from the European Saccharomyces cerevisiae Archives for Functional Analysis (Euroscarf) [30]. In Y03825 the Ca^2+^ antiporter gene *VCX1* (YDL128w, [31]) is replaced by the *kanMX4* gene that in yeast provides a geneticin (G418, Sigma G8168) resistant phenotype. BY4741n is the isogenic and haploid parental strain (Euroscarf Y00000). Yeast cells were transformed by the DMSO-based method [32]. Yeast were grown on complete YPD (yeast-peptone) medium with 2% (w/v) D-glucose, or minimal synthetic medium (SM) with 2% (w/v) glucose or 2% (w/v) galactose as carbon sources, and strain-specific required nutrients and 400 μg/ml G418 [33].

### Apoaequorin luminescence assay

Apoaequorin assays were adapted from Miseta *et al*[34]. pSEY8 vector (pAEQ with a *URA3* selection marker) expresses the soluble calcium-binding protein aequorin [29]. Cells carrying pSEY8 and the pGREG505 recombinant vectors were grown in SD and 2% (w/v) glucose until they reached exponential growth to 0.5-0.6 OD_600_/ml. The cultures were then washed twice in SD medium and grow a further four hours in SD with 2% (w/v) galactose to induced gene expression. Ten OD_600_ units of cells were resuspended in 180 μl of test medium –TM- (0.17% (w/v) YNB, 2 mM EGTA, 40 mM MES-Tris, pH 6.5) after two washes with the same medium. To equilibrate cytosolic aequorin with its allosteric group, 20 μl of 0.6 mM coelenterazine (Fluka Biochemica 07372) dissolved in methanol were added and the cell suspension incubated for 30 min at room temperature. Cells were then washed twice in TM. The final pellet was resuspended in 1 ml of TM and allowed to equilibrate for 30 min at room temperature. After detecting the baseline light emission 1 ml of 1 M of CaCl_2_ was added. Final concentrations of CaCl_2_ were 50 mM or 500 mM in 2 ml total volume. For the multi-well plate experiments 0.1 OD_600_ were plated in 0.1 ml volumes and 1 M of the divalent cation salts (CaCl_2_, MgCl_2_ or MnCl_2_) were added in 0.1 ml to make 0.5 M final concentrations in 0.2 ml total volume. Light emission was collected from the 2 ml assays with a CliniLumat Berthold luminometer at intervals of 2 seconds or from the 0.2 ml multi-well assays with a LumiStarOmega luminometer (BMG Labtech) plate reader at 0.5 seconds intervals. Where indicated the samples were incubated with dimethylsulphoxide (DMSO) alone or with Bafilomycin A_1_ dissolved in DMSO (1 μM final concentration, Sigma B1793) or KB-R7943 dissolved in DMSO (Calbiochem 420336) for 30 minutes at room temperature prior to the addition of CaCl_2_.

### Indirect immunofluorescence assay (IFA)

Yeast membranes were extracted and purified as in Fisher *et al*[35]. Indirect immunofluorescence in yeast cells was performed as in Stearman *et al*[36]. Polyclonal IgG antibody Anti-PfCHA (0.568 mg/ml) was produced in rabbit by GenScript (Piscataway, NJ, USA) against a 14 aa synthetic peptide representing positions 73-86 in the amino terminal sequence of PfCHA and used at 1:100 dilutions. The Anti-actin antibody was purchased from Sigma (A2066) and used at 1:500 dilutions. The secondary antibody anti-rabbit IgG-FITC was also purchased from Sigma (F0382) and used at 1:1000 dilutions. Cells were mounted with VectaShield HardSetTM mounting medium (Vector Labs Burlingame, CA, USA) and observed in a confocal microscope Zeiss Axiovert 200 M (L5M 5Pascal laser modules). Propidium iodide (Fluka 70335) was used for nuclei localization at 10 μg/ml room temperature for 30 minutes after treating with 100 μg/ml of RNaseA for 30 minutes.

## Results

### PfCHA restores mobilization of cytoplasmic Ca^2+^ in *Saccharomyces cerevisiae vcx1Δ*

Yeast *vcx1Δ* has a significant defect in the rapid Ca^2+^ sequestration response to high cytosolic levels of this cation [34]. Recombinant pGREG505 vectors carrying PfCHA or yeast *VCX1* genes as well as the empty vector control were transformed into *vcx1Δ*. For the detection of cytosolic Ca^2+^ by bioluminescence, these strains were subsequently transformed with a vector carrying the apoaequorin gene (pSEY8-AEQ). Positive colonies were selected for growth in the absence of leucine and uracil and in the presence of 400 μg/ml of geneticin in SD medium. The presence of the recombinant vectors was verified and their RNA expression monitored by reverse transcription and PCR amplification (Additional file 1). Expectedly, there is a relative low expression of the heterologous PfCHA gene in the *vcx1Δ* mutant in comparison to the native yeast *VCX1* gene. Truncated gene transcription is a known phenomenon in yeast expressing AT-rich *P. falciparum* genes [37] and this is evident in the differential amplifications for fragments in comparison to full-length *VCX1* and PfCHA genes (Additional file 1B). Nonetheless the bioluminescence assays as presented below showed that these levels of expression were sufficient to detect functional protein in the yeast *vcx1Δ* background.

**Figure 1:**
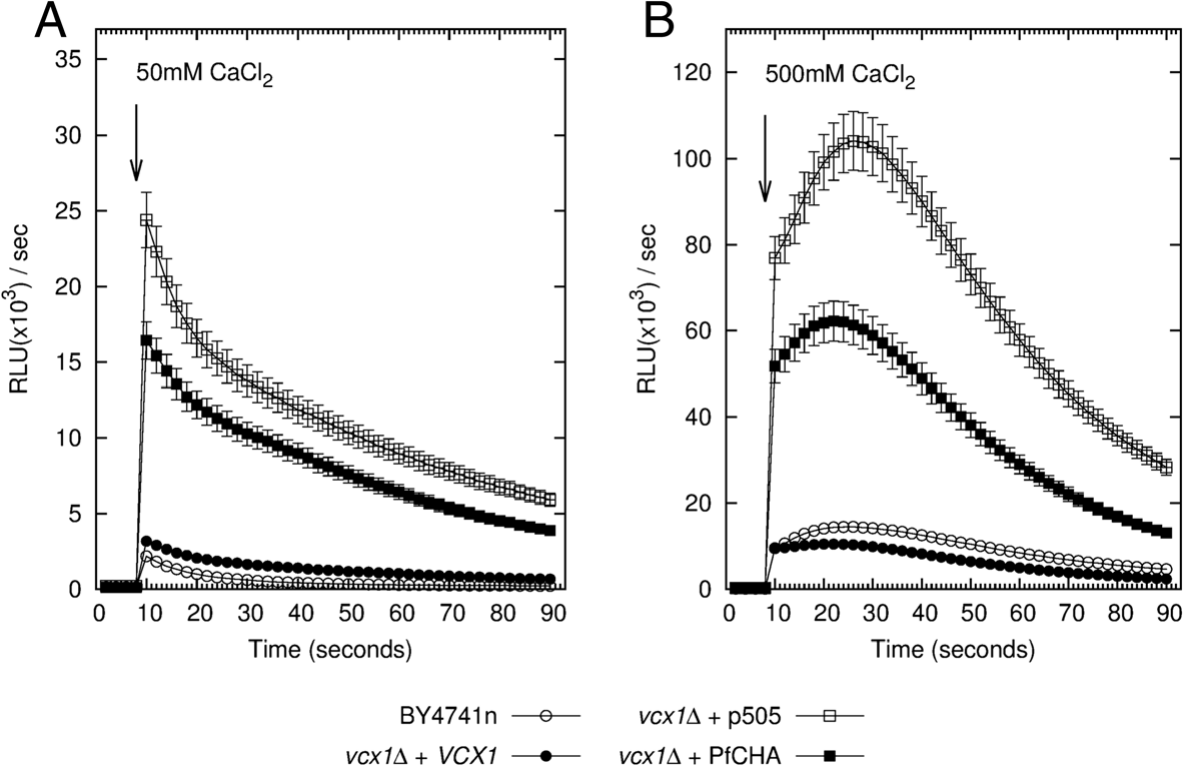
**PfCHA rescues yeast *Saccharomyces cerevisiae *Ca**^**2+**^**/H**^**+ **^**antiporter mutant. A.** Apoaequorin bioluminescence of cytosolic Ca^2+^ following exposure to extracellular Ca^2+^. Light emission was initially recorded for 8 seconds in low Ca2+ medium. CaCl_2_ was subsequently added to 50 mM final concentration in 2 ml total volume. The luminescence recorded for 90 seconds is shown. BY4741n is the haploid isogenic yeast parental strain, *vcx1*Δ + p505 is the *vcx1*Δ knock-out carrying the pGREG505 plasmid only, *vcx1*Δ + *VCX1* and *vcx1*Δ + PfCHA are the same knock-out strain but carrying recombinant plasmids with the homologueous *VCX1* gene or the *Plasmodium falciparum* PfCHA gene, respectively. **B.** Apoaequorin luminescence measurements following a challenge with 500 mM extracellular CaCl2. Addition of CaCl2 is indicated by the arrows. Data represent means and standard deviations of three experiments (n=3).

The medium used to process cells for the apoaequorin bioluminescence assays contains 2 mM EGTA to reduce extracellular Ca^2+^ concentration in the test medium - TM (~6 μM [34]). Four different samples were examined: the parental isogenic and haploid strain BY4741n, the *vcx1Δ* mutant containing the pGREG505 vector only (*vcx1Δ* + p505), *vcx1Δ* carrying the recombinant pGREG505-*VCX1* (*vcx1Δ* + *VCX1*), and *vcx1Δ* carrying the recombinant vector pGREG505 with PfCHA (*vcx1Δ* + PfCHA). All four strains responded with a sharp elevation in cytosolic Ca^2+^ levels when 50 mM CaCl_2_ was added (Figure 1A).

Before the Ca^2+^ shock the luminescence of the samples were registered for 8 seconds to establish the basal line. As expected *vcx1Δ* mutant showed the highest peak of cytosolic Ca^2+^ level and the reference BY4741n strain presented the lowest signal with a 10-fold difference between them. Mutant *vcx1Δ* cells expressing the homologue *VCX1* closely followed the Ca^2+^ levels of BY4741n. Significantly, *vcx1*Δ cells expressing the heterologous PfCHA also followed the extrusion of cytosolic Ca^2+^ after the initial peak. Although as anticipated less efficiently than the homologue *VCX1* gene (Figure 1A). The difference in the areas under the curve (AUC) between the cytosolic levels of Ca^2+^ in the mutant *vcx1Δ* carrying the plasmid control and the *vcx1Δ* carrying the recombinant plasmid with PfCHA was significant (p < 0.005, Student’s two-tailed test). Replacing D-galactose with D-glucose, and thereby inhibiting the transcription of genes under the *GAL1* promoter of the pGREG505 vector, impaired the expression of both *VCX1* and PfCHA (Additional file 2). The phenotype of the *vcx1*Δ mutant and the different levels of rescue by *VCX1* or PfCHA detected as cytosolic levels of Ca^2+^ were more apparent (p *<*0.0001 and p *<*0.001, respectively, Student’s two-tailed test) when even higher concentrations (500 mM) of CaCl_2_ were applied as external Ca^2+^ (Figure 1B).

**Figure 2:**
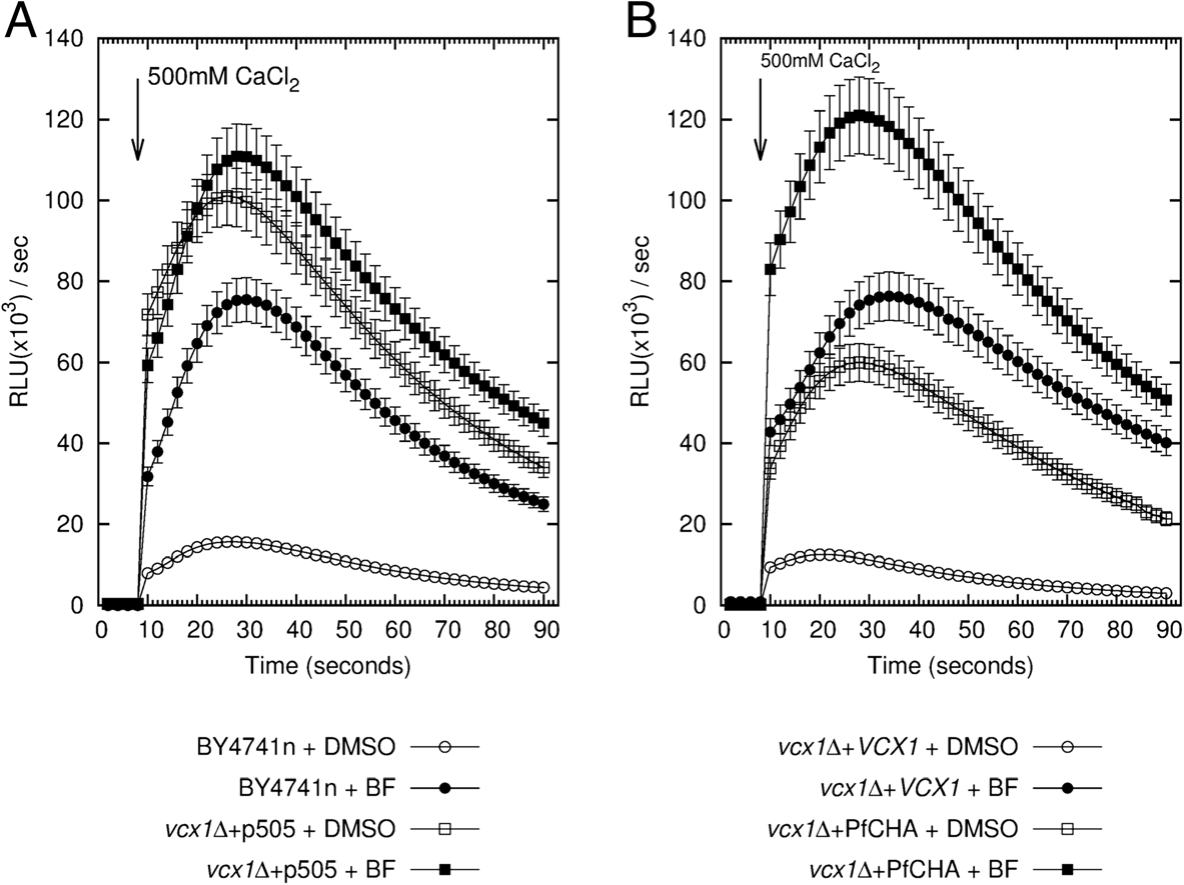
**PfCHA Ca2+/H + antiporter function in yeast is inhibited by bafilomycin A1. **Cytoplasmic Ca^2+^ dependent apoaequorin luminescence was measured following pre-incubation with the V-type ATPase inhibitor bafilomycin A1 (BF). Inhibitor-free controls contained concentrations of solvent (DMSO) equivalent to the solvent present in the BF sample. **A.** Data from the parental BY4741n strain and the *vcx1Δ* mutant carrying the plasmid only (*vcx1Δ *+ p505). **B.***vcx1Δ* carrying the yeast *VCX1* (*vcx1Δ *+* VCX1*) or PfCHA (*vcx1Δ *+ PfCHA). Time of the addition of CaCl_2_ is indicated by the arrows. Data represent means of three experiments (n = 3).

Ca^2+^/H^+^ antiporters can be inhibited by interfering with the transmembrane H^+^- gradient [38] and thus PfCHA was expected to be sensitive to bafilomycin A_1_ a vacuolar-specific V-type ATPase inhibitor. Following a pre-incubation with bafilomycin A_1_ (1 mM, 30 minutes) Ca^2+^ shock experiments were carried out as described above with 500 mM CaCl_2_. For all the strains, bafilomycin A_1_ treatment resulted in a reduced ability of the yeast cells to recover from the Ca^2+^ challenge (Figure 2). The reference strain BY4741n showed a three-fold increased cytosolic Ca^2+^ peak (Figure 2A), while the *vcx1Δ* mutant peak response was slightly but not significantly higher in the presence of bafilomycin A_1_ (Figure 2A) than the DMSO control. As with the strain carrying the yeast Ca^2+^/H^+^ exchangers (p *<*0.0001, Student’s two-tailed test), treatment with bafilomycin A_1_ also affected the ability of PfCHA-carrying yeast cells to recover from the Ca^2+^ challenge (p *<*0.001, Student’s two-tailed test) (Figure 2B). Taken together, the described phenotype rescue experiments carried out with the yeast *S. cerevisiae vcx1Δ* mutant are indicative of the functional expression of the heterologous PfCHA. Consistent with its expected mechanism of transport PfCHA is dependent on a proton membrane gradient sensitive to the vacuolar-specific inhibitor bafilomycin A_1._

### Further functional studies in a scalable format

**Figure 3:**
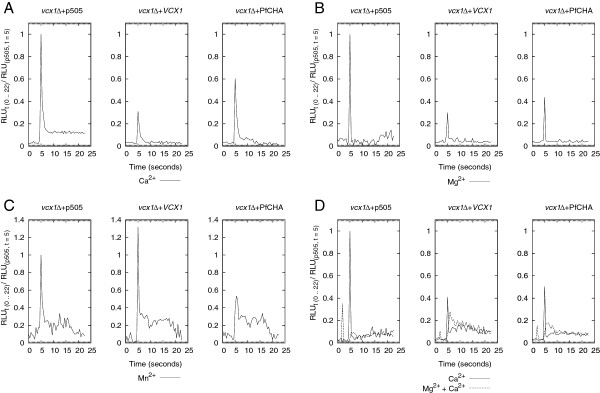
**Divalent cation transport activities in multi-well plates. A-C.** Apoaequorin bioluminescence for cytosolic Ca^2+^ , Mg^2+^ and Mn^2+^, respectively. Cell cultures were distributes in 0.1 ml volumes in 96-well plates at 0.1 OD_600_ densities. A bolus of 0.1 ml 1 M of CaCl_2_, MgCl_2_, or MnCl_2_ was added at 5 seconds and the relative light units (RLUs) were recorded every 0.5 second for 60 seconds. The signals for experimental group were normalized against the RLU of the 5 second point for that group (RLU_t (0 .. 22)_/RLU_t = 5_) and the signals for the first 22 seconds are presented. D. Dual magnesium and calcium transport. Cell cultures in 0.08 ml volumes were distributed in 96-well plates at 0.1 OD_600_ density. MgCl_2_ was added (1M, 0.02 ml) at 2 seconds followed by 0.1 ml of 1M CaCl_2_ at 5 seconds. RLUs were normalized as in **A–C.** Data presented is representative of four experiments for **A–C** and three experiments for **D.**

Yeast cultures of *vcx1Δ* carrying the pGREG505 vector only (*vcx1Δ* + p505) or the recombinant vectors pGREG505–*VCX1* (*vcx1Δ* + *VCX1*) and pGREG505-PfCHA (*vcx1Δ* + PfCHA) were processed for apoaequorin assays as described in Methods. The parental strain BY4741n was not used for assays in the multi-well plates as the mutant carrying the homologue *VCX1* gene behaved equivalently in the Ca^2+^ loading assays presented above. In 96-well plates yeast cells were distributed in 0.1 ml per well containing a number of cells equivalent to 0.1 OD unit (OD_600_). After establishing the base line for 5 seconds Ca^2+^, Mg^2+^ or Mn^2+^ were injected as 1 M chloride salts on to the selected wells (0.1 ml) to render 0.5 M final concentrations. The luminescence signals were recorded for 60 seconds at intervals of 0.5 seconds. The luminescence peaks were sharper and the area under the curves was smaller than those originated by the larger cell volumes and densities used previously with equivalent final concentrations of Ca^2+^ (Figure 1B). The dissipation of free cytosolic Ca^2+^ by Vcx1p and PfCHA was significant (p *<*0.001 and p *<*0.01, respectively, Student’s two-tailed test) by comparison to the vector only control demonstrating the functionality of the assays in the multi-well format (Figure 3A). Furthermore, the effective movement of free Mg^2+^ (Figure 3B) and Mn^2+^ (Figure 3C) from the cytosol by PfCHA was also apparent and significant (p *<*0.01). Noteworthy, the initial functional characterization of PfCHA as a Ca^2+^ and Mn^2+^ transporter did not present direct transport activity of Mg^2+^ by PfCHA when expressed in *X. laevis* oocytes [20]. However, a lower affinity of PfCHA for Mg^2+^ was inferred from competition studies against ^45^Ca^2+^ transport. Here, the yeast expression system seems to substantiate the evidence of Mg^2+^ being a substrate for PfCHA-mediated transport. On the other hand, the yeast Vcx1p antiporter is known to lack Mn^2+^ transport [39] which is in agreement with the observed lack of suppression of the Mn^2+^ cytosolic peak in the cells expressing the gene product of *VCX1* (Figure 3C).

The effective sequestration of cytosolic Mg^2+^ was corroborated when cells pre-loaded with Mg^2+^ (0.2 M final concentration) had a subsequently bolus of Ca^2+^ (0.5 M final concentration) (Figure 3D). The rationale behind this assay takes into account the fact that Mg^2+^ and Ca^2+^ share their binding sites in aequorin. Aequorin is known to bind other divalent cations besides Ca^2+^ via one or more of its three EF-hand Ca^2+^-binding sites and Mg^2+^ has been shown to compete for the same sites that Ca^2+^ occupies when bound to aequorin [40,41]. Thus pre-loading with Mg^2+^ will be expected to reduce any signal from a subsequent Ca^2+^ injection if the former has not been effectively sequestered from the cytosol. Therefore, the magnitude of the luminescence signal from Ca^2+^ will be in proportion to the effective sequestration of Mg^2+^ from cytoplasm. As observed in Figure 3D the injection of Mg^2+^ at 2 seconds showed the expected signal in response to the presence of this divalent cation in the cytosol. When the injection of Ca^2+^ followed at 5 seconds, the three different cell groups presented a delayed luminescence peaks in comparison to the cells that were not pre-loaded with Mg^2+^ (Figure 3D). The magnitude of the luminescence peak generated by Ca^2+^ seems to reflect rather accurately the capacity for transport of cytoplasmic Mg^2+^ (Figure 3D). The Ca^2+^-dependent signal was respectively 12 times, 1.5 times and 3.5 times on average lower in the cells pre-loaded with Mg^2+^ than those that were not for *vcx1Δ* + p505*, vcx1Δ* + *VCX1* and *vcx1Δ* + PfCHA. Magnesium was then more effectively cleared in the cells carrying PfCHA followed by the yeast vcx1p antiporter and with a significantly lack of transport for Mg^2+^ in the knock-out stain.

PfCHA as well as Vcx1p, however, sequestered Ca^2+^ at slower rates when cells had been pre-loaded with Mg^2+^. Yeast Vcx1p dissipated the Ca^2+^ signal 13 times slower on average (means 0.32 RLUs/second *vs* 0.025 RLUs/second) in the presence of Mg^2+^, with PfCHA rates of Ca^2+^ clearance falling to 63 times on average (means 0.44 RLUs/second *vs* 0.007/second) significantly slower when in the presence of Mg^2+^. Clearance rates for Ca^2+^ were calculated from the highest point to the first point of the plateau for each curve (Ca^2+^ only versus Ca^2+^ after Mg^2+^ load). The delayed response to cytoplasmic Ca^2+^ is interpreted as the time required for Ca^2+^ to displace Mg^2+^ from the aequorin EF-hand Ca^2+^-binding sites, and the slower clearance rates as the inhibitory effects of Mg^2+^ on the transport of Ca^2+^ by Vcx1p and PfCHA. Interestingly, by comparison PfCHA seems to be a more proficient transporter of Mg^2+^ given that a higher proportion was cleared from cytoplasm by PfCHA (Ca^2+^ peak lower in cells loaded with Mg^2+^). Also, PfCHA seems to have a higher affinity for Mg^2+^ than the yeast Vcx1p since the rate of Ca^2+^ sequestration of cytosolic Ca^2+^ in cells loaded with Mg^2+^ was most affected in the cells expression PfCHA. Pre-loading with Mn^2+^ was less informative due to the overlapping patterns with the signal generated by Ca^2+^ (data not shown). This is observed as the result of Mn^2+^ occupying a EF-hand Ca^2+^-binding site different from the two sites that Ca^2+^ binds in aequorin [23,24].

### Inhibitory assays with KB-R7943

**Figure 4:**
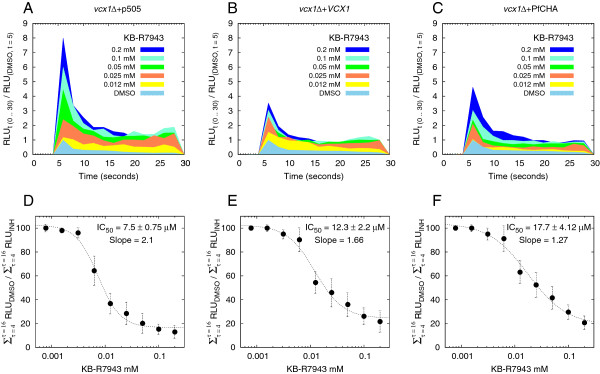
**Calcium transport inhibition assays with KB-R7943. A – C.** Ca^2+^ - dependent luminescence at increasing concentrations of KB-R7943. Cells were incubated for 30 minutes in 2% (v/v) DMSO (concentration of solvent in the culture with the highest concentration of KB-R7943) or in increasing concentrations of KB-R7943. The profiles for the first 30 seconds are presented for 12.5 μM, 25 μM, 50 μM, 100 μM and 200 μM of KB-R7943. CaCl_2_ (1 M, 0.1 ml) was added at 5 seconds to wells containing 0.1 ml of cells (0.1 OD_600_) and the signals recorded for 60 seconds. RLUs were normalized against the RLU of the 5 second point for that group ( RLU_t (0 .. 30)_/RLU_t = 5_ ). Data represent the averages of four experiments. **D – E.** Inhibitory concentrations for KB-R7943. Inhibitory assays were performed with nine concentrations at two-fold dilutions from 0.2 mM KB-R7943. Y axis: the areas under the curve (AUC) from 4 to 16 seconds for the DMSO samples were divided by the AUC for the different concentrations of KB-R7943 in each experimental group and multiplied by 100 to express the fractions as percentages. The data were fitted and the IC_50_ and slopes inferred with the following equation: f(x) = A2 + (A1 – A2)/(1 + (x/L) ^S^ ). Where “A2” is the limit to the left of the sigmoid (highest signal, lowest concentration), “A1” is the limit to the right (lowest signal, highest concentration), “L” is the concentration equivalent to half of the signal of the left limit (IC_50_), and “S” is the slope. Data represents the averages and standard deviations of four different experiments.

In the absence of specific Ca^2+^/H^+^ antiporter inhibitors KB-R7943 was used to illustrate an inhibitory assay for PfCHA using the yeast expression system. KB-R7943 is an inhibitor of Na^+^/Ca^2+^ antiporters [42-44] although it inhibits more avidly members of the canonical transient receptor potential channel (TRPC) family [45]. More recently KB-R7943 was used in the functional characterization of PfCHA expressed in *X. laevis* oocytes [20] where it reduced by approximately half the uptake of Ca^2+^ at 20 μM. Here yeast cultures were incubated for 30 minutes at room temperature in several concentrations of KB-R7943 after the cells had been treated with coelenterazine as in Methods. The effect of this inhibitor on the sequestration of cytosolic Ca^2+^ was apparent in all three experimental groups of the *vcx1Δ* knock-out strain carrying the empty vector pGREG505 (*vcx1Δ* + p505) or expressing *VCX1* (*vcx1Δ* + *VCX1*) and PfCHA (*vcx1Δ* + PfCHA) (Figures 4A–4C). The luminescence signals increased in proportion to the concentration of KB-R7943 in all three groups: higher peak at injection time and larger areas under the curve (AUC) throughout the detection time of 60 seconds. Next, the observed luminescence patterns were translated to inhibitory dose–response curves. The ratios of the AUC (between 4 and 16 seconds) of the luminescence signals from the DMSO controls over the AUC for the signals from the KB-R7943 samples were calculated and expressed as percentages for each of the three experimental groups (Figures 4D–4F). All three *vcx1Δ* yeast mutants, carrying the vector only as well as the yeast carrying Vcx1p or the *Plasmodium* PfCHA, had their mobilization of cytosolic Ca^2+^ inhibited by KB-R7943. The average concentrations (n = 4) at which their transport was inhibited by 50% (the AUC of the luminescence signal had double with respect to the DMSO sample) was calculated to be 7.5 μM, 12.5 μM and 17.5 μM of KB-R7943 for *vcx1Δ* + pGREG505, *vcx1Δ* + *VCX1* and *vcx1Δ* + PfCHA, respectively (Figures 4D–4F). The higher sensitivity of the knock-out strain to this inhibitor in comparison to either of the *vcx1Δ* expressing the Ca^2+^/H^+^ antiporters from yeast or *Plasmodium* could be explained if the *S. cerevisiae* TRPC homologue Yvc1p, a vacuolar Ca^2+^- and mechanosensitive TRP cation channel, mediates the rapid initial response for sequestration of Ca^2+^ in *vcx1Δ* cells. TRP channels are targets of KB-R7943 and have been shown to have their current blocked by IC_50_ in the range of 0.46–1.38 μM of this inhibitor. These data therefore demonstrate the proof of concept that the yeast system using the *vcx1Δ* knock-out and apoaequorin (aequorin + coelentarazine) as a divalent cation reporter delivers a suitable screening setting for high throughput search for molecules with inhibitory activity against *P. falciparum*’s Ca^2+^/H^+^ antiporter PfCHA.

### Cytolocalization of PfCHA in *Saccharomyces cerevisiae*

**Figure 5:**
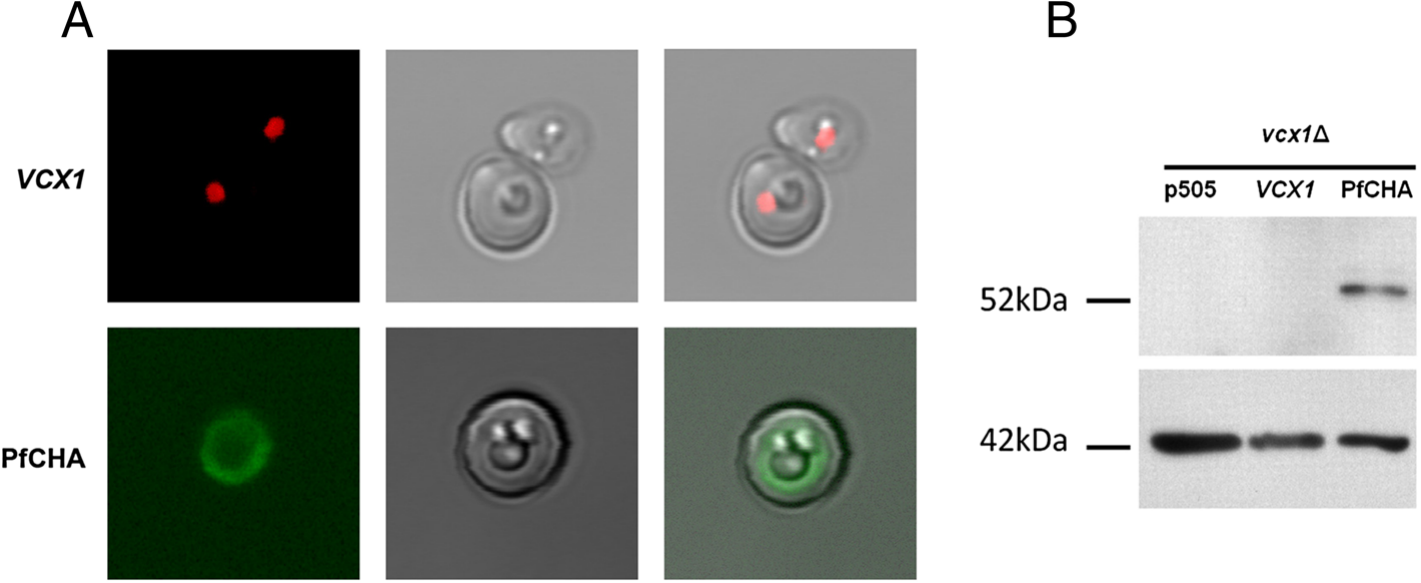
**PfCHA protein localizes to the yeast vacuole membrane. A.** Indirect immunofluorescence assay with Anti-PfCHA in yeast *vcx1Δ* expressing the recombinants pGREG505-*VCX1* and pGREG505-PfCHA. Bright field, dark field, and the merge in columns from left to right. Top panels show a sample from cells carrying pGREG505-*VCX1* and stained with propidium iodide for staining of the nucleus in addition to the primary Anti-PfCHA. Bottom panels shown FITC fluorescence localizing around the yeast tonoplast of *vcx1Δ* cells carrying pGREG505-PfCHA and probed with Anti-PfCHA (1:100). **B.** Anti-PfCAX Western Blot. Top panel shows the signal given by Anti-PfCHA on the yeast membrane extracts. The expected molecular size for PfCHA is approximately 49kDa. Samples are labelled as the yeast *vcx1Δ* mutant carrying the empty pGREG505 vector (p505), or the recombinant pGREG-*VCX1* or pGREG-PfCHA vectors. Bottom panel show the control with Anti-actin antibody. Molecular mass markers as indicated.

Using a primary antibody (Anti-PfCHA) raised against a 14 aa synthetic peptide representing residues 73-86 of PfCHA, indirect immunolocalization assays were performed in the transformed yeast. Yeast *vcx1Δ* mutants carrying pGREG505-PfCHA showed a fluorescent signal derived from the vacuole (tonoplast) (Figure 5A, bottom panels) while the control samples using the strain carrying pGREG505-*VCX1* (Figure 5A, top panels) failed to generate the same fluorescent signal. The specificity of the Anti-PfCHA used here was assessed probing blots with membrane-enriched fractions of the same *vcx1Δ* mutant expressing the recombinants *VCX1* or PfCHA (Figure 5B). These observations in addition to the functional data presented above, indicate that in contrast to the mitochondria localization of PfCHA in *P. falciparum* and in the plasma membrane when expressed *X. laevis*[20], in *S. cerevisiae* the same protein is sorted to the vacuole membrane.

## Discussion

A transient rise in cytosolic free Ca^2+^ generated when influx temporarily exceeds efflux usually functions as an intracellular signal. Calcium-proton exchangers are low-affinity cytosolic export systems that coupled to the thermodynamically downhill exchange flux of H^+^ dissipate cytosolic increases of Ca^2+^[21]. PfCHA is part of the CAXs (for CAtion eXchangers) group of integral membrane proteins that transport Ca^2+^ energized by the electrochemical gradient established by proton pumps such as H^+^-ATPases and H^+^-pyrophosphatases [17]. Accordingly, CAX proteins had previously been located in acidic compartments in plants [17] and yeast [21] where they sequester calcium from the cytoplasm. In *P. falciparum* however, the CAX homologue PfCHA seems to localize and perform Ca^2+^ (as well as Mn^2+^) exchange in the mitochondrion of this parasite [20]. In this organelle PfCHA is proposed to mediate the extrusion of peaks of Ca^2+^ from the matrix when the mitochondrion is going through transient Ca^2+^ overloads [20].

In order to detect functional PfCHA in *S. cerevisiae*, the apoaequorin luminescent response to divalent cations was used as a reporter system to follow cytosolic levels of Ca^2+^, Mg^2+^ and Mn^2+^[29]. Apoaequorin bioluminescence assays in yeast have been applied in studies of cellular Ca^2+^ homeostasis [34,46], including the functional characterization of heterologous Ca^2+^ transporters such as the *A. thaliana* Ca^2+^/H^+^ antiporters (*AtCAX*) [47]. Here, PfCHA was shown to rescue the ability to mediate the dissipation of cytosolic peaks of Ca^2+^, Mg^2+^ and Mn^2+^ in the yeast *vcx1Δ* mutant and that this activity was sensitive to inhibition of the H^+^ transmembrane gradient by a vacuolar-ATPase inhibitor as well as the Na^+^/H^+^ antiporter and TRP channels inhibitor KB-R7943. The levels of cytosolic Ca^2+^, Mg^2+^ and Mn^2+^ reported by the apoaequorin luminescence show apparent and significant cytosolic dissipation of all three of these divalent cations by PfCHA in *vcx1Δ* cells. Taken together, the functional data and immunolocalization point at PfCHA to behave as a divalent cation/H^+^ exchanger that in yeast functions as a vacuolar membrane transporter. As a consequence of the role of the proton gradient in the function of this type of calcium exchanger potential inhibitors of PfCHA will have to be tested for their own capacity to inhibit proton pumps.

In yeast it is estimated that at least 90% of the total 1.5-4 mM of cellular Ca^2+^ is sequestered within the yeast vacuole in stable complexes (e.g. with polyphosphates) as well as free Ca^2+^[48] allowing the cell basal cytosolic levels to be kept around 50-80 nM. The accumulation of vacuolar Ca^2+^ in yeast is maintained through the complementary action of Vcx1p [48,49] and the Ca^2+^ ATPase Pmc1p [50]. As with other CAX proteins Vcx1p is a low affinity high capacitance facilitative Ca^2+^ transporter that responds to sudden increments of the cytosolic Ca^2+^ by rapid sequestration of this cation into the vacuole in exchange for two protons down a proton gradient (vacuolar pH 5.5-6.2 *vs* cytoplasmic pH 7.0) [38,51].

It is noteworthy that in *S. cerevisiae,* cells have to be exposed to seemingly excessive concentrations of these cations (50 mM to 500 mM external levels of their chloride salts) in order to reach measurable cytosolic transient elevations of these ions. When exposed to 50 mM *S. cerevisiae* cytosolic levels of Ca^2+^ are measured to be in the region of 250-290 nM (basal concentrations 50–80 nM) that increases further to 320-350 nM when the external Ca^2+^ is raised to 400 mM [34]. The *vcx1Δ* mutant undergoes transient elevations slightly higher with cytosolic calcium concentrations of approximately 400 nM and 510 nM when in the presence of 50 mM and 400 mM of Ca^2+^, respectively [34]. Coincidentally, these cytosolic concentrations of Calcium are far from the 2 mM reported as the Km of PfCHA for Ca^2+^[20], or the Km for Ca^2+^ reported for other CAX proteins (up to 25μM) [21]. Nonetheless, the rates of divalent cations transported by PfCHA effectively dissipated 40 – 55% of the cytosolic transient elevations in the *vcx1Δ* background (Figures 1-Figure 3).

The localization of PfCHA to the yeast vacuole is not altogether surprising as this organelle seems to be the default localization for foreign proteins in *S. cerevisiae*. In *S. cerevisiae* proteins are sorted to the vacuole by at least four different pathways [52]: (i) sorting of vacuolar proteins *vs* cell surface proteins in the early stages of the secretory pathway, (ii) endocytic engulfing of material from the plasma membrane, (iii) cytoplasm-to-vacuole targeting pathways that do not transit the early stages of the secretory pathway, and (iv) the *de facto* inheritance of vacuolar material by daughter cells during cell division. However, in *S. cerevisiae* membrane proteins lacking motifs or otherwise protein structure information for cellular localization are predominantly delivered to the vacuole membrane [53,54]. Moreover, membrane proteins have been shown to be delivered by default to the yeast vacuole when high levels of protein expression – overexpression – are achieved. The localization of membrane proteins depends also on the length of their transmembrane domains [53], it is not possible to know *a priori* the destination of a membrane transporter in a heterologous expression system. In the yeast expression system as reported here it is speculated that PfCHA is sorted by a default mechanism to the yeast vacuole [55].

In eukaryotic cell hosts, the yeast *S. cerevisiae* is by far the most advantageous gene expression system since it is relatively inexpensive to culture and it is the most genetically tractable eukaryotic system with excellent recombinant DNA tools. Additionally, recent advances seeking to overcome the difficulties intrinsic to the expression of foreign proteins in yeast, particularly membrane proteins, have rendered transgenic strains with altered ribosomal content that seems to tolerate and much improve heterologous gene overexpression [56,57]. The successful expression of PfCHA in *S. cerevisiae* and its localization to the yeast vacuole permits a very tractable system for further functional and pharmacological studies as exemplified here in multi-well whole-cell cation transport and inhibitory assays.

## Conclusions

Yeast offers a cost-effective and user-friendly gene expression system for further functional studies and pharmacological screens of proteins from *P. falciparum*. Particularly membrane transporters such as PfCHA that are still very seldom successfully expressed in heterologous hosts. Moreover, the bioluminescence assay for the detection of cytoplasmic Ca^2+^ as well as Mg^2+^ and Mn^2+^ lends itself to high-throughput and quantitative settings.

## Abbreviations

PfCHA: *Plasmodium falciparum* Ca^2+^/H^+^ antiporter; VCX1: *Saccharomyces cerevisiae*; Ca^2+^/H^+^ antiporter; RLU: Relative Light Units.

## Competing interests

The authors declare that they have no competing interests.

## Authors’ contributions

JES-S designed the study and performed experiments. JES-S and GAB wrote the initial draft of the manuscript. SAW and GAB edited and revised the manuscript, and provided materials and equipment. All authors read and approved the final manuscript.

## Supplementary Material

Additional file 1 **PfCHA and *VCX1 *RNA yeast expression. **A. The expression of *Plasmodium* PfCHA as well as yeast *VCX1* was observed by reverse transcription PCR using total RNA from the reference strain BY4741n, the *vcx1Δ* mutant carrying the expression plasmid only (p505) and the same mutant carrying the recombinant plasmid with either *VCX1* or PfCHA. Forward and reverse primer position and sequences: PfCHA (64 - 488nt) (5’- AAAAATGTGCCCCCTATGAA, 5’-TCCATTAAATTACCAAACGTAGCA), *VCX1* (269 - 666nt) (5’-GTAACACCATTGGGGGACTG, 5’-ATGGCTCCCTAGCTGGAAAT), and yeast *S. cerevisiae* actin *YFL039c* (130 - 533 nt) (5’- ATGGTCGGTATGGGTCAAAA, 5’- ATTCTCAAAATGGCGTGAGG). B. Real-time PCR using total RNA from the *vcx1Δ* mutant carrying recombinant plasmids with *VCX1* or PfCHA following the amplification of either a fragment or the full gene for *VCX1–*666 bp and *VCX1*-1236 bp respectively. Likewise for the PfCHA (488 bp fragment or entire gene of 1321 bp). Primers for total length genes as in Methods. Evidently, RNA levels of PfCHA as full size gene are present around three-fold lower than its homologous counterpart *VCX1* while the difference in the levels of shorter (truncated) transcribed fragments is only half that difference. Click here for file

Additional file 2 **Phenotypic rescue of yeast *Saccharomyces cerevisiae vcx1Δ *inhibited in presence of glucose. **Conditions and strains as in Figure 1A except that the culture medium contained D-glucose instead of D-galactose which suppresses the activity of the *GAL1* promoter present in pGREG505. Click here for file
