# Supplementary figures and images for: A yeast expression system for functional and pharmacological studies of the malaria parasite Ca2+/H+ antiporter
Source: Malar J. 2012 Aug 1;11:254. doi: 10.1186/1475-2875-11-254 (PMC3488005; doi:10.1186/1475-2875-11-254)

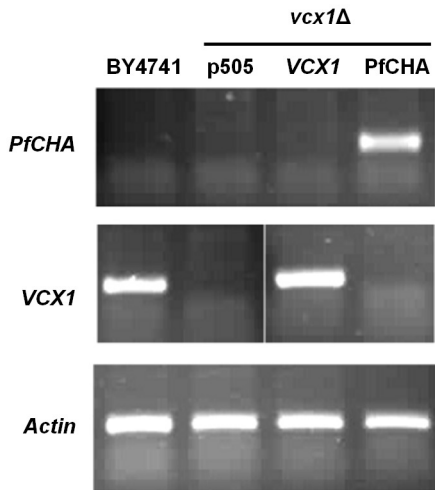

**A**

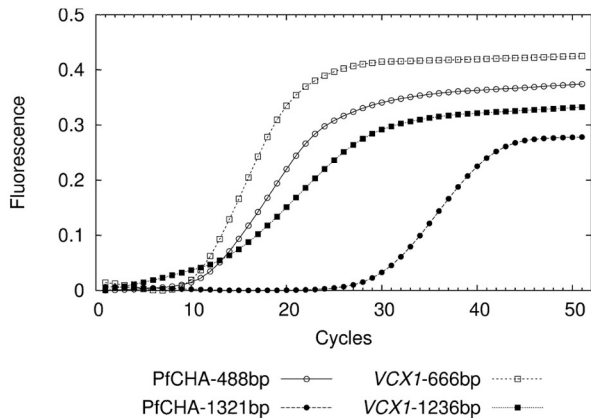

**B**

Additional Figure 1

Supplement: Additional file 1 — PfCHA and VCX1 RNA yeast expression. A. The expression of Plasmodium PfCHA as well as yeast VCX1 was observed by reverse transcription PCR using total RNA from the reference strain BY4741n, the vcx1Δ mutant carrying the expression plasmid only (p505) and the same mutant carrying the recombinant plasmid with either VCX1 or PfCHA. Forward and reverse primer position and sequences: PfCHA (64 - 488nt) (5’- AAAAATGTGCCCCCTATGAA, 5’-TCCATTAAATTACCAAACGTAGCA), VCX1 (269 - 666nt) (5’-GTAACACCATTGGGGGACTG, 5’-ATGGCTCCCTAGCTGGAAAT), and yeast S. cerevisiae actin YFL039c (130 - 533 nt) (5’- ATGGTCGGTATGGGTCAAAA, 5’- ATTCTCAAAATGGCGTGAGG). B. Real-time PCR using total RNA from the vcx1Δ mutant carrying recombinant plasmids with VCX1 or PfCHA following the amplification of either a fragment or the full gene for VCX1–666 bp and VCX1-1236 bp respectively. Likewise for the PfCHA (488 bp fragment or entire gene of 1321 bp). Primers for total length genes as in Methods. Evidently, RNA levels of PfCHA as full size gene are present around three-fold lower than its homologous counterpart VCX1 while the difference in the levels of shorter (truncated) transcribed fragments is only half that difference. [file 1475-2875-11-254-S1.pdf]

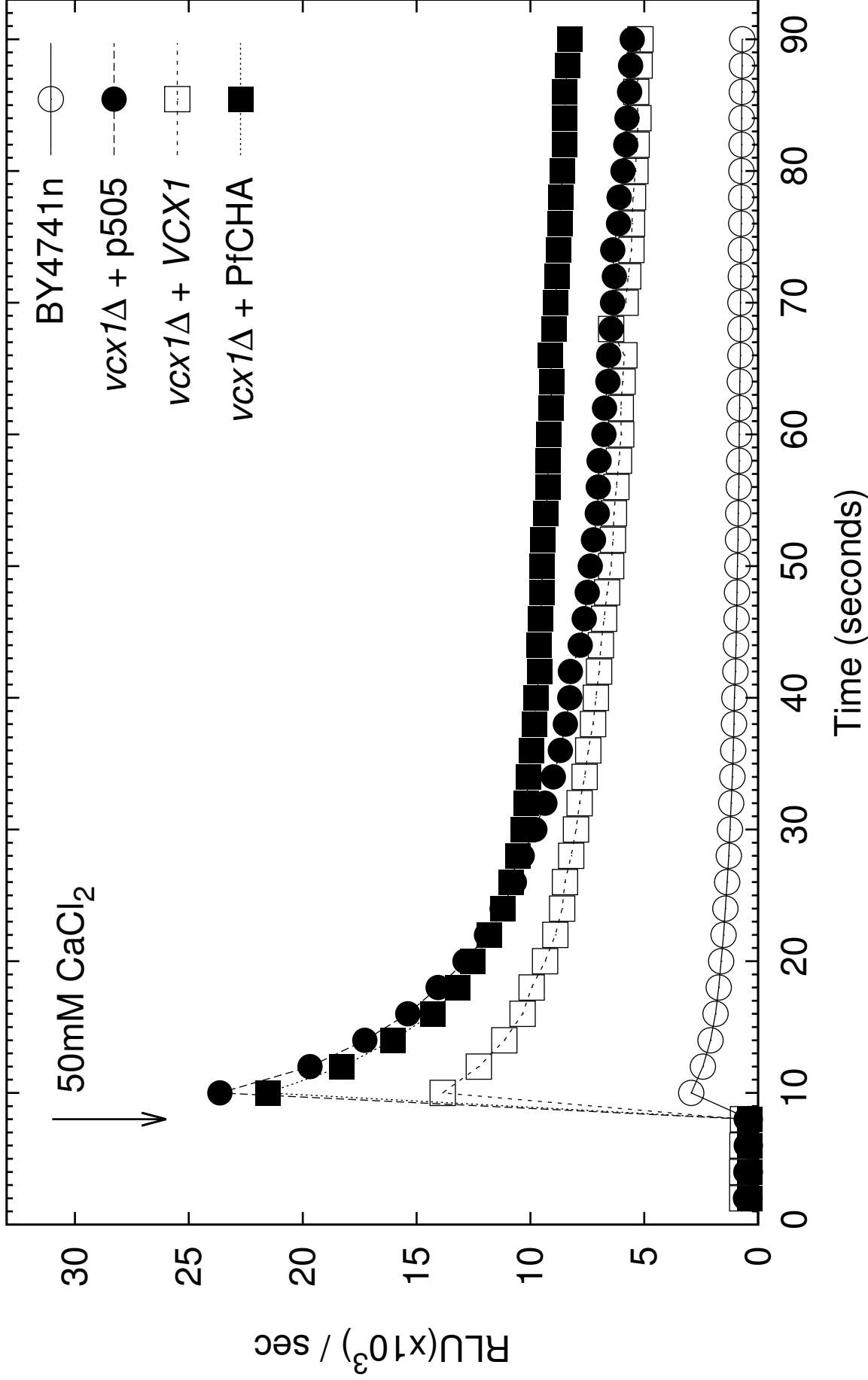

Additional Figure 2

Supplement: Additional file 2 — Phenotypic rescue of yeast Saccharomyces cerevisiae vcx1Δ inhibited in presence of glucose. Conditions and strains as in Figure 1A except that the culture medium contained D-glucose instead of D-galactose which suppresses the activity of the GAL1 promoter present in pGREG505. [file 1475-2875-11-254-S2.pdf]
